# Fast 3-Breath-Hold 3-Dimensional Tagging Cardiac Magnetic Resonance in Patients with Hypertrophic Myocardial Diseases: A Feasibility Study

**DOI:** 10.1155/2016/3749489

**Published:** 2016-02-28

**Authors:** Yasuo Amano, Fumi Yamada, Hidenobu Hashimoto, Makoto Obara, Kuniya Asai, Shinichiro Kumita

**Affiliations:** ^1^Department of Radiology, Nippon Medical School, 1-1-5 Sendagi, Bunkyo-ku, Tokyo 113-8603, Japan; ^2^Department of Cardiology, Toho University Omori Hospital, 6-11-1 Omori-Nishi, Tokyo 143-8541, Japan; ^3^Philips Electronics Japan, 2-13-37 Konan, Minato-ku, Tokyo 108-8507, Japan; ^4^Department of Cardiology, Nippon Medical School, 1-1-5 Sendagi, Bunkyo-ku, Tokyo 113-8603, Japan

## Abstract

Tagging CMR has been established as the standard reference for measurement of myocardial strain. The current 2D tagging technique requires multiple breath-holds to cover the whole heart and cannot show the 3D motions of the left ventricle. We performed fast 3-breath-hold 3D tagging with localized tagging preparation and complementary spatial modulation of magnetization in 10 patients with hypertrophic myocardial diseases and 6 normal volunteers. The left wall motion was observed at any view angle, which allowed for the identification of regional and global hypokinesis using the fast 3D tagging. Although a decrease in the circumferential strain and LGE were observed at the basal septum in hypertrophic cardiomyopathy, they were not located together in each patient. In hypertensive heart disease, the decrease in circumferential strain was observed more widely than LGE, and the summed strain of all segments was significantly decreased. The decrease in strain and LGE were observed diffusely in cardiac amyloidosis. In conclusion, fast 3-breath-hold 3D tagging is feasible for the regional and global strain analysis. The location of reduced circumferential strain is not necessarily the same as that of LGE and is related to the global cardiac function in patients with hypertrophic myocardial diseases.

## 1. Background

Myocardial hypertrophy is induced by genetic mutations, storage diseases, or reaction to hypertension, aortic valvular disorders, or obstruction of the left ventricular outflow tract. Myocardial hypertrophy may lead to a decrease in coronary reserve flow, which is related to adverse cardiac events [1, 2].

Cardiac magnetic resonance (CMR) is used to quantify regional and global cardiac function and myocardial thickness and mass and to detect myocardial scarring [3–5]. In particular, late gadolinium enhancement (LGE) CMR is valuable for the identification of the myocardial scarring associated with hypertrophic myocardial diseases including hypertrophic cardiomyopathy (HCM), hypertensive heart disease (HHD), and amyloidosis, and LGE is strongly related to serious complications and the prognosis of the patients [6–9]. Tagging CMR is another useful method that quantifies the regional or global strain related to myocardial fiber architecture, estimates cardiac dyssynchrony, and identifies subclinical systolic impairment [10–13]. In HCM and HHD, the regional heterogeneity of the strain, decrease in circumferential strain, or abnormal apical rotation is observed using tagging CMR [13, 14].

Although tagging CMR has been established as the standard reference for measurement of myocardial strain and motion, current 2-dimensional (2D) tagging CMR requires multiple breath-holds to cover the whole heart. The 2D imaging technique cannot show the 3-dimensional (3D) motions of the left ventricle. Ryf et al. [15] developed 3D tagging with complementary spatial modulation of magnetization (CSPAMM). A detraction of 3D tagging is its lengthy scan time. Rutz et al. [16] developed fast 3D tagging by using line tagging in the 3 spatial directions, the spatial localized pulse for the second tagging preparation, and echo-planar imaging (EPI) readout. The fast 3D tagging allows for the whole heart to be imaged with 3D tagging with only 3-breath-holds and was applied to 5 patients with myocardial infarction. However, to our knowledge, there have been no previous studies to evaluate the myocardial strain with the fast 3D tagging technique in patients with nonischemic, hypertrophic myocardial diseases with myocardial stiffness and regional scarring.

In the present study, we sought to evaluate the feasibility of fast 3-breath-hold 3D tagging for the assessment of the circumferential strain in patients with hypertrophic myocardial diseases. We also compared the locations with the abnormal strain with those of LGE.

## 2. Methods

### 2.1. Subjects

Ten patients with a maximum wall thickness ≥ 15 mm were recruited between June 2014 and August 2015. They were 9 men and 1 woman ranging in age from 35 to 92 years (68.2 ± 16.1 years). They comprised 5 patients with HCM, 3 with HHD, and 2 with cardiac amyloidosis. One patient with HHD had associated myocardial infarction. The diagnosis of the hypertrophic myocardial diseases was made by endomyocardial biopsy or from a combination of family history of HCM, past history, electrocardiogram (ECG), and imaging studies [6–9]. For comparison, 6 healthy male volunteers (age: 30–61 years; 42.0 ± 14.4) underwent the fast 3D tagging. This study followed our institutional ethical guidelines given by the IRB.

### 2.2. CMR Protocol

**Figure 1:**
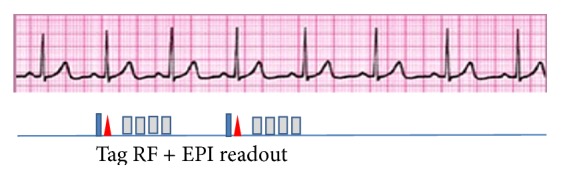
Imaging sequence for fast 3-breath-hold 3D tagging. The line tagging (blue box and red triangle) was applied in 3 orthogonal directions. The second tagging preparation (red triangle) was the spatial localized pulse, and its use and echo-planar imaging readout (gray box) in combination with CSPAMM allow for fast 3-breath-hold 3D tagging of the whole heart.

CMR studies were performed using a 3.0 T unit (Achieva, Philips Healthcare, Best, The Netherlands). A cardiac phased-array coil was used for signal reception, and vector ECG was used for cardiac gating. After localizer scanning, short-axis 2D cine steady-state free precession was performed with the following image parameters: repetition time (TR), 4.1 ms; echo time (TE), 2.0 ms; flip angle, 45–55°; in-plane resolution, 1.6 × 1.7 mm^2^; slice thickness, 8 mm with a 2 mm gap; and 20–24 phases per cardiac cycle. Thereafter, fast 3-breath-hold 3D tagging was performed with imaging parameters as follows: TR, 6.5 ms; TE, 3.0 ms; flip angle, 17°; EPI factor (i.e., echo train length), 7; in-plane resolution, 4.4 × 4.4 mm^2^; slice thickness, 8.8 mm; slice partition, 14; and 24 phases per cardiac cycle. A ramped flip angle was used to prevent the tag fading. The line tagging with 8 mm spacing was applied in 3 orthogonal directions. The second tagging preparation was the spatial localized pulse, which allows for a half field-of-view without a wrapping-around artifact (Figure 1) [15–17]. The position of the diaphragm during 3-breath-holds was maintained identically using a navigator technique with a 15 mm window and a correction factor of 0.6 [18]. These imaging techniques in combination with CSPAMM induced fast 3-breath-hold 3D tagging of the whole heart (3D Tag, GyroTools, Zurich, Switzerland) [11, 15, 16]. Approximately 12 min after the injection of gadolinium-based contrast agent at a dose of 0.10–0.15 mmol/kg, LGE CMR was performed with the imaging parameters as follows: TR, 10 ms; TE, 2.9 ms; flip angle, 15°; pixel size, 1.8 × 1.2 mm^2^; and slice thickness, 10 mm. The inversion time to null the signal from the normal myocardium was adjusted for each patient.

### 2.3. Image Analysis

Ejection fraction (EF), maximum wall thickness, and myocardial mass of the left ventricle were acquired from the cine data. The presence of myocardial LGE was determined when its mean signal intensity was above 6 SD of the mean signal intensity of the nullified myocardium. In cases of amyloidosis, the LGE was identified as global and diffuse LGE as it was in previous studies [9, 19].

**Figure 2:**
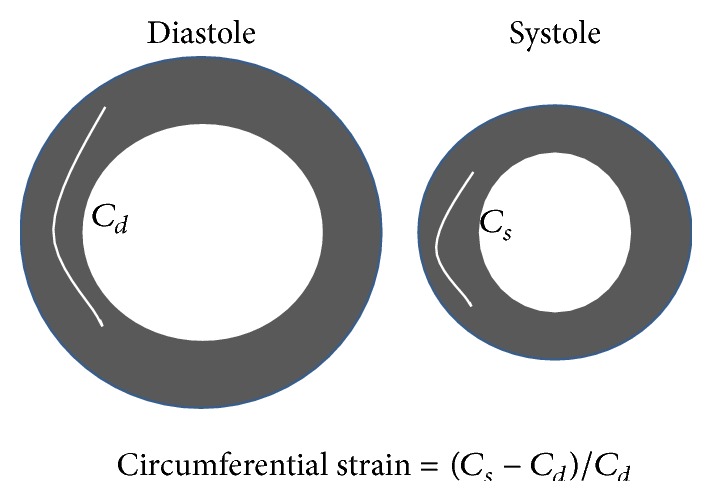
How circumferential strain is obtained is shown. *C*_*d*_ and *C*_*s*_ represent circumferential length at diastole and systole related to the strain, respectively.

One radiologist with 18-year experience of CMR analyzed the fast 3D tagging using dedicated software (TagTrack 3D, GyroTools, Zurich, Switzerland). The harmonic phase method was used to track the myocardial wall motion after the correction of magnetic inhomogeneity and smoothing the boundaries with a bandpass filter [16]. The midwall contour at the basal, middle, and apical levels was divided according to a 16-segment model from the American Heart Association [20]. The circumferential strain was defined as the fractional change (%) in the myocardial length in the direction tangential to the epicardial wall [13, 14] (Figure 2).

First, we assessed the technical feasibility of fast 3-breath-hold 3D tagging based on the success rates of the scanning, reconstruction of cine 3D surface representation, and acquisition of the quantitative regional strain data. Second, we compared the circumferential strain of the patients with those of the healthy volunteers at the 16 myocardial segments. In the present study, we defined the decrease in strain as being 20% less than the mean strain value of the normal volunteers. In addition, the comparison for circumferential strain was made between the normal volunteers and HCM patients at each segment, or the summed strain of all 16 segments was compared between the normal volunteers and patients with HCM, HHD, or cardiac amyloidosis. An unpaired* t*-test was used to assess the difference, and *P* < 0.05 was defined as statistically significant. Third, the circumferential strain was compared with the presence of LGE at each myocardial segment. A Fisher test was used to assess the relationship between the circumferential strain and LGE when appropriate, and *P* < 0.05 was considered significant.

## 3. Results

**Figure 3:**
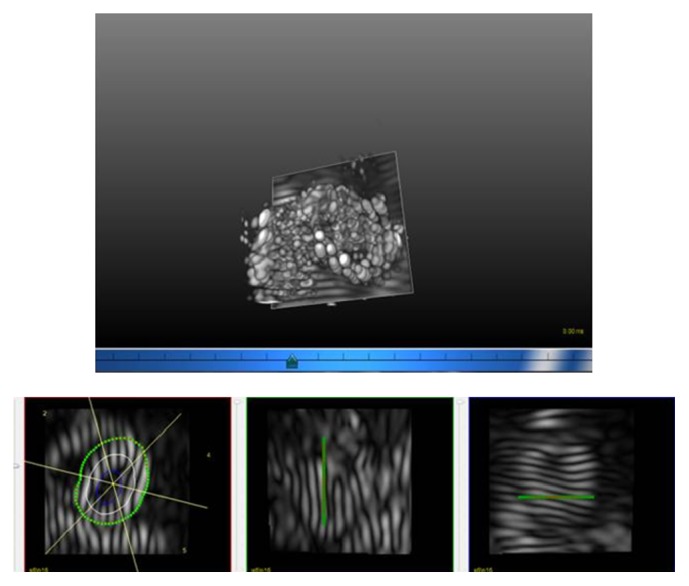
Reconstructed 3-dimensional image and source images in the 3 orthogonal directions are shown.

Fast 3-breath-hold 3D tagging was completed in all of the 10 patients and 6 healthy volunteers. The 3D surface representation in cine mode was successfully generated in all of the subjects. The circumferential strain was estimated in all of the myocardial segments of all subjects enrolled (Figure 3, Supplemental video in Supplementary Material available online at http://dx.doi.org/10.1155/2016/3749489, and Table 1).

Table 1 shows the circumferential strain in patients and controls. Because the normal strain values were below zero as expected [10–13], the decrease in strain was defined as that above zero or that being 20% less than the absolute value of the normal mean strain. In HCM, the circumferential strain reduced at the inferior septal segment at the basal level and apical septal segment. However, there were no significant differences in the strain between the normal volunteers and patients with HCM at any segments (*P* > 0.14). In HHD, the circumferential strain predominantly decreased at the mid septal and posterior segments and apical segments. The circumferential strain was also reduced at the basal anterior segment. The circumferential strain was decreased in 10 and 12 of the 16 segments in the 2 patients with cardiac amyloidosis. The summed strain of all segments was significantly decreased in patients with HHD (−8.3 ± 7.6; *P* = 0.010), but not in those with HCM (−17.3 ± 11.8) or those with amyloidosis (−11.0 ± 6.8), compared with the normal volunteers (−14.1 ± 18.4).

LGE was observed in HCM, whereas the segments with LGE were not identical to those with reduced circumferential strain in each patient. There was no relationship between the reduced circumferential strain and LGE in HCM patients (*P* = 0.73). In HHD, the segments with reduced circumferential strain were observed more widely than those with LGE. LGE was expectedly observed diffusely in cardiac amyloidosis.

## 4. Discussion

Fast 3-breath-hold 3D tagging successfully provided 3D surface representation in cine mode and circumferential strain data. Compared with the normal volunteers, the patients with HCM or HHD showed reduced circumferential strain at several myocardial segments. The segments showing reduced circumferential strain were not identical to those with LGE in HCM, and the reduced strain was distributed more widely than LGE in HHD. The reduced strain and LGE were observed extensively in patients with cardiac amyloidosis. Thus, the fast 3-breath-hold 3D tagging may be feasible for detection of the circumferential strain decrease, which is not necessarily associated with myocardial scarring in patients with hypertrophic myocardial diseases.

The scan time for 3D tagging was reduced by using line tagging in the 3 spatial directions, a spatial localized pulse for the second tagging preparation, and an EPI readout [16]. The position of the diaphragm during breath-hold was maintained identically during the 3-breath-holds using navigator technique [18]. Thereby, fast 3-breath-hold 3D tagging successfully provided 3D surface representation in cine mode and the regional circumferential strain in all of the subjects.

The circumferential strain deteriorated even in HCM patients with a preserved EF. A hypertrophied basal septum and apical septum tended to show a decrease in circumferential strain in HCM, which indicates the dominant changes in the septal myocardial architecture [21]. The segments with reduced circumferential strain were not concordant with those showing LGE. This result was not consistent with that of the previous study [22], partly because the patchy midwall LGE might not affect the circumferential strain in HCM. Aletras et al. [23] indicated that LGE does not necessarily explain reduced strain, which is estimated using displacement encoding with stimulated echoes, in HCM. When summing the strain at all segments, we did not find the reduction in circumferential strain in patients with HCM. Thus, the 3D tagging identified only regional wall abnormality in HCM patients with preserved EF.

In the 3 patients with HHD and low EF, the reduced strain was distributed more widely than LGE. The mid septal-to-posterior segments showed reduced circumferential strain in our study. These segments tend to have LGE in HHD patients with congestive heart failure [7, 9]. Foell et al. [24] showed a reduction in the radial and longitudinal strain of the mid posterior areas. Therefore, fast 3D tagging can identify the changes in myocardial architecture changes that preceded LGE in patients with HHD. In addition, the 3D tagging showed a decrease in the summed strain of all myocardial segments in patients with HHD, all of whom had decreased EF.

In cardiac amyloidosis, a decrease in circumferential strain and LGE were observed diffusely, as expected [9, 19].

There are several limitations to this study. First, the study population was small. The statistical analyses were affected by this limitation. In addition, almost all of the subjects were men because HCM and HHD are predominantly observed in men. Thus, the present results might not be possible to extrapolate the findings to female patients. Second, the volunteers were younger than the patients. Because there were some segments with reduced circumferential strain, subclinical myocardial disorders could not be excluded in the volunteers. The “normal” heterogeneity of the strain was also considered for the lower values of the strain [25]. Third, we defined the decrease in the strain as being 20% less than the mean strain value of the volunteers; this definition is somewhat arbitrary. Fourth, we investigated the whole left ventricular motion only in 3D surface representation and analyzed the regional strain only at 3 levels, because the volume of data acquired with 3D fast tagging was enormous relative to software capability. The development of sophisticated software to analyze 3 types of myocardial strain in all of the left ventricular regions (e.g., 6 segments × 14 slices × 24 cardiac phases) is warranted. Last, we did not compare the strain and histology. Endomyocardial biopsy provided a diagnosis of HHD or cardiac amyloidosis, whereas the biopsy was conducted without referring to the 3D fast tagging. Moreover, the circumferential strain data was acquired at the midwall, while the histological samples were acquired from the myocardium close to the ventricular cavity.

## 5. Conclusions

Fast 3-breath-hold 3D tagging allows for regional and global wall strain analysis. The location of reduced circumferential strain was not necessarily the same as that of LGE in patients with hypertrophic myocardial diseases. Fast 3D tagging may be feasible for the detection of myocardial strain changes which are not necessarily associated with myocardial scarring in the patients with HCM or HHD.

## Supplementary Material

3D cine representation generated from fast 3-breath-hold 3D tagging in a patient with HHD and global decrease in myocardial motion.

## Figures and Tables

**Table 1:** Circumferential Strain (%) Estimated by 3-Breath-Hold 3D Tagging CMR in Patients with Hypertrophic Myocardial Diseases and Normal Volunteers.

segment	1	2	3	4	5	6	7	8	9	10	11	12	13	14	15	16	EF (%)
HCM	−14.0	−20.0	*28.0*	−10.0	−22.0	*23.0 *	*6.8 *	−33.0	−25.0	−15.0	−25.0	*17.0 *	***−6.7***	**−24.0 **	**−27.0 **	−23.0	62.5
	−22.0	***−15.0***	**−14.0 **	−7.9	−20.0	−11.0	**−35.0 **	***−13.0***	−19.0	−23.0	**−25.0 **	**−26.0 **	*−15.0 *	−25.0	−16.0	−25.0	79.7
	−14.0	−22.0	*−10.0 *	−13.0	*−5.4 *	−14.0	−19.0	*−14.0 *	−24.0	−12.0	−9.4	−14.0	*−12.0 *	*−15.0 *	−21.0	−27.0	67.1
	***−10.0***	**−22.0 **	*6.0 *	−12.0	−22.0	−22.0	−12.0	−18.0	**−23.0 **	*−5.3 *	−16.0	−25.0	*−14.0 *	−22.0	−31.0	*−9.8 *	54.0
	−16.8	−28.1	**−13.6 **	−17.9	−21.5	−10.6	−22.6	**−17.6 **	*−16.9 *	−23.9	−31.7	−24.3	−35.7	−28.3	−18.2	−19.4	56.8
mean	−15.4	−21.4	−0.7	−12.2	−18.2	−6.9	−16.4	−19.1	−21.6	−15.8	−21.4	−14.5	−16.7	−22.9	−22.6	−20.8	64.0

HHD	*−1.6 *	**−17.0 **	**−39.0 **	*−6.5 *	−13.0	***−1.6***	−16.0	*−9.9 *	*−17.0 *	*9.0 *	***−4.2***	**−20.0 **	*−6.7 *	*−7.5 *	*−12.0 *	***−6.7***	22.7
	*−10.0 *	−20.0	*−8.6 *	−6.9	*−5.3 *	−14.0	6.9	*−9.2 *	*−10.0 *	*3.8 *	*−4.0 *	−12.0	*−5.2 *	−19.0	*−12.0 *	*−7.6 *	13.8
	*7.9 *	*−14.1 *	*−7.1 *	*8.0 *	*−9.4 *	−8.7	−14.0	*−9.1 *	*−18.0 *	−20.0	*10.0 *	−16.0	*−8.4 *	*−12.0 *	*−11.0 *	*−6.9 *	33.5

Amyl	**−22.0 **	***−10.0***	***−11.0***	***−4.8***	***−10.0***	**−15.0 **	**−18.8 **	***−9.9***	***−17.6***	***−3.9***	**−18.5 **	**−18.2 **	***−13.5***	**−22.0 **	***−10.2***	***−17.1***	31.0
	*0.3 *	*−7.6 *	***0.6***	**−9.8 **	***−2.4***	*−1.9 *	*−1.9 *	***−1.6***	*−14.0 *	**−10.4 **	***−3.0***	***−0.9***	*−0.7 *	−21.9	***−12.7***	−20.4	60.8

Normal	−14.3	−20.3	−15.4	−8.4	−12.9	−9.5	−9.2	−18.5	−23.5	−11.0	−6.0	−8.6	−25.5	−22.7	−17.7	−23.7	NA

Italic: abnormal circumferential strain (%), bold: segments with late gadolinium enhancement (LGE). HCM: hypertrophic cardiomyopathy, HHD: hypertensive heart disease, Amyl: cardiac amyloidosis, Normal: normal volunteers, NA: not available. The mean value of circumferential strain was shown in normal volunteers. In HCM, the segments with LGE were not identical to those with reduced strain in each patient. In HHD with low ejection fraction, the segments with reduced strain were observed more widely than those with LGE. The reduction in strain and LGE were observed diffusely in cardiac amyloidosis.

## Abbreviations

CMR: Cardiac magnetic resonance
CSPAMM: Complementary spatial modulation of magnetization
ECG: Electrocardiogram
HCM: Hypertrophic cardiomyopathy
HHD: Hypertensive heart disease
LGE: Late gadolinium enhancement
3D: Three-dimensional.
